# Imaging Markers of Multiple System Atrophy and Their Association With Disease Severity: A Cross-Sectional Study

**DOI:** 10.7759/cureus.67896

**Published:** 2024-08-27

**Authors:** Santosh Kumar Dash, Nitish Kamble, Albert Stezin, Ravi Yadav, M Netravathi, Jitender Saini, Pramod Pal

**Affiliations:** 1 Neurology, Kalinga Institute of Medical Sciences, Bhubaneswar, IND; 2 Neurology, National Institute of Mental Health and Neurosciences, Bengaluru, IND; 3 Clinical Sciences, Centre for Brain Research, Indian Institute of Science, Bengaluru, IND; 4 Neuroimaging and Interventional Radiology, National Institute of Mental Health and Neurosciences, Bengaluru, IND

**Keywords:** hot cross bun sign, multiple system atrophy (msa), msa-c cerebellar type, unified multiple system atrophy rating scale (umsars), msa-p parkinsonian type

## Abstract

Background

Multiple system atrophy (MSA) is a rare, adult-onset neurodegenerative disorder marked by autonomic failure, parkinsonism, and cerebellar ataxia, with subtypes classified as parkinsonian (MSA-P), cerebellar (MSA-C), and autonomic (MSA-A). This study aims to identify MRI biomarkers for MSA and their correlation with disease severity.

Methodology

A total of 30 patients with probable MSA (20 MSA-C, 10 MSA-P) aged 45-65 years were studied. Motor and non-motor symptoms were assessed using the Unified Multiple System Atrophy Rating Scale (UMSARS), and all patients underwent 3T MRI brain imaging. Data analysis was performed using SPSS version 22 (IBM Corp., Armonk, NY, USA) with Spearman’s correlation for clinical-imaging correlations.

Results

The mean age of the study population was 54.43 years, with a male predominance (56.7%). The most common symptoms included gait ataxia (43.3%) and urinary dysfunction (96.7%), with orthostatic symptoms in 33.3%. Moderate disease severity was observed, with mean UMSARS scores of 15.9 (Part 1) and 16 (Part 2), showing no significant subtype differences. MRI revealed abnormalities in all patients, predominantly cerebellar atrophy (90%). The “hot cross bun” (HCB) sign was seen in 75% of MSA-C patients, but none of MSA-P patients showed the same. The HCB sign was significantly correlated with severity in MSA-C (USMSARS-4). Putaminal signs were less frequent and slightly more prevalent in MSA-P, without significant clinical-imaging correlation.

Conclusions

This study reinforces the critical role of MRI biomarkers in the diagnosis of MSA patients. Notably, the HCB sign exhibited a significant association with clinical severity in MSA-C patients, while such correlation was absent in MSA-P cases.

## Introduction

Multiple system atrophy (MSA) is a rare neurodegenerative disease of adults characterized by severe autonomic failure, parkinsonism, and/or cerebellar ataxia [1]. It is divided into parkinsonian (MSA-P) and cerebellar (MSA-C) subtypes based on the predominant motor symptom, and sometimes MSA-A, where autonomic symptoms are the predominant symptoms [2]. The average annual incidence of MSA is between 0.6 and 1 case per 100,000 population per year, with an equal incidence among males and females [3]. Based on the new diagnostic criteria of the Movement Disorder Society 2022 for the diagnosis of clinically established MSA, one imaging marker on an MRI is a mandatory requirement [4]. Atrophy of the middle cerebellar peduncle (MCP), the cerebellum, and the pons are more common in the MSA-C subtype. Similarly, the MSA-P subtype more frequently exhibits widespread cerebral atrophy, putamial hyperintensity, and putaminal atrophy [5]. The diagnosis of MSA remains challenging due to a lack of specific biomarkers. Although MRI markers have shown promise in aiding the diagnosis and monitoring of MSA progression, their correlation with disease severity remains unclear. The objective of this study was to identify the various imaging biomarkers in MSA and correlate them with disease severity in patients with MSA. This will significantly contribute to enhancing our understanding of the neuroimaging features of MSA and its subtypes.

## Materials and methods

Study setting and ethics

This cross-sectional study was conducted in the Department of Neurology at the National Institute of Mental Health and Neurosciences (NIMHANS), Bengaluru, Karnataka, India. All study participants provided written informed consent, and the Ethics Committee approved the study under the protocol IEC/No. 3.02/3-11-2015.

Study participants

The study cohort included 30 patients aged between 45 and 65 years, who were diagnosed with clinically probable MSA. Patients demonstrating clinical features of parkinsonism, gait ataxia, and autonomic symptoms were recruited from both outpatient and inpatient neurology departments, following a thorough examination by neurologists and meeting the second consensus diagnostic criteria of MSA [6]. Exclusion criteria encompassed patients with contraindications for brain MRI. Furthermore, all participants underwent a thorough evaluation to exclude alternative causes of cerebellar and cerebral atrophy, including drug-related, metabolic, or toxic factors. Positive cases for these alternative causes were subsequently excluded from the study. Sociodemographic data, encompassing age at presentation, age of onset, gender, and duration of illness, were systematically collected using a predefined proforma.

Clinical evaluation

Participants underwent a comprehensive clinical examination to assess tremors, bradykinesia, rigidity, postural instability, levodopa responsiveness, and cerebellar symptoms such as gait or limb ataxia and speech problems. The severity of MSA was evaluated using the Unified Multiple System Atrophy Rating Scale (UMSARS) with global disability rated on Part 4 of the UMSARS scale ranging from 1 to 5, indicating the severity of the disability [7]. Autonomic and various non-motor symptoms, including orthostatic hypotension and urinary symptoms, were also assessed.

Brain MRI

Brain MRI scans (3T) were obtained for all patients and controls following a predefined protocol using the Philips Achieva 3-T MRI system (Philips Healthcare, Best, Netherlands). The imaging sequences acquired included T1-weighted, T2-weighted, fluid-attenuated inversion recovery, and diffusion-weighted imaging, with a senior neuroradiologist evaluating all MRI images, who was not blinded to the clinical characteristics of MSA patients.

Statistical analysis

Clinical data were analyzed using SPSS Statistics version 22 (IBM Corp., Armonk, NY, USA). Continuous variables were expressed as mean and standard deviation (SD), while categorical variables were presented as frequency and percentage. The chi-square test was used for categorical variables, with p-values <0.05 considered statistically significant. Spearman’s correlation coefficient helped determine the correlation between clinical severity and MRI findings.

## Results

Clinicodemographic features

The study enrolled 30 patients with probable MSA. There were 10 MSA-P and 20 MSA-C patients. The mean age of the patients was 54.43 ± 5.86 years. For MSA-P patients, the mean age was 55.7 ± 5.4 years, whereas for MSA-C patients, it was 53.8 ± 6.0 years (p = 0.71). Of all patients, 56.7% were males and 43.3% were females. Males had a higher incidence of MSA-C (1.5:1) than females. The gender distribution was equal in MSA-P.

The mean age at symptom onset in the patient group was 51.8 ± 5.9 years. In MSA-C, the mean age at onset was 51 ± 6.4 years, and in MSA-P, it was 53.3 ± 4.7 years. There was no significant difference between the two groups (p = 0.33). The mean duration of illness was 2.6 ± 1.2 years. In MSA-P, the mean duration was 2.4 ± 1.3 years, and in MSA-C, it was 2.7 ± 1.2 years, without any statistically significant difference between the two groups (p = 0.49). Among all patients, 20 (67%) were diagnosed with MSA-C and 10 (33%) with MSA-P. The majority of patients were diagnosed with MSA-C, with gait ataxia being the most common initial symptom seen in 43.3% of cases. Other initial symptoms included slowness of activities, asymmetric tremors of hands, urinary disturbances, speech disturbances, and incoordination of hands. These findings suggest a varied presentation of symptoms in patients with MSA. Rapid eye movement sleep behavior disorder (RBD) as an initial symptom was present in 6.7% of cases before their motor symptoms. Gait imbalance and asymmetric onset tremors of the hands were the most common initial symptoms seen in 65% of MSA-C and 50% of MSA-P patients.

Autonomic dysfunction was present in most individuals. Urinary abnormalities were the most common autonomic dysfunction seen in 29 (96.7%) patients. Urgency was observed in 8 (28%) patients, while urinary frequency was observed in 19 (65%) individuals. Two (7%) patients had urinary incontinence.

In total, 10 (33.3%) patients experienced orthostatic symptoms. The mean reduction in systolic blood pressure (SBP) was 17.1 ± 11.8 mmHg, and the mean diastolic blood pressure (DBP) drop was 7.9 ± 4.5 mmHg. The mean DBP drop and SBP decline in MSA-C were 8.2 ± 5.3 mmHg and 17.5 ± 11.9 mmHg, respectively. The mean decline in DBP was 7.5 ± 2.3 mmHg, and the mean drop in SBP was 16.4 ± 12.2 mmHg in MSA-P. There was no significant difference in the change in blood pressure between the two subtypes (p = 0.56).

The UMSARS was utilized to evaluate the clinical severity. The mean Part 1 and Part 2 UMSARS scores for the patients were 15.9 ± 8.4 and 16 ± 8.2, respectively. The Part 1 score for MSA subtypes was 15.4 ± 5.0 in MSA-P and 16.2 ± 9.8 in MSA-C. In MSA-P and MSA-C, the Part 2 scores were 16.8 ± 7.7 and 16.9 ± 9.6, respectively. These were not statistically significant. The median global disability score (UMSARS-4) of MSA patients was 2 (1-5), with an equal score of 2 (1-5) in both MSA subtypes. All clinicodemographic features are shown in Table 1.

**Table 1:** Demographic and clinical characteristics of patients with MSA. DBP = diastolic blood pressure; MSA = multiple system atrophy; RBD = rapid eye movement behavior disorder; SBP = systolic blood pressure; UMSARS = Unified Multiple System Atrophy Rating Scale

	Total patients	MSA-P	MSA-C	P-value
Clinical characteristics				
Number of patients (N)	30	10	20	-
Gender (female:male ratio)	43.3%:56.7%	1:1	1.5:1	-
Age (mean ± SD, years)	54.43 ± 5.86	55.7 ± 5.4	53.8 ± 6.0	0.71
Age at onset (mean ± SD, years)	51.8 ± 5.9	53.3 ± 4.7	51 ± 6.4	0.33
Symptoms				
Gait ataxia (% of cases)	43.3%	50%	65%	0.30
Urinary disturbances (% of cases)	96.7%	88%	96%	0.52
Orthostatic symptoms (% of cases)	33.3%	20%	40%	0.46
RBD (as the initial symptom, % of cases)	6.7%	-	-	-
Drop in SBP (mean ± SD, mmHg)	17.1 ± 11.8	16.4 ± 12.2	17.5 ± 11.9	0.56
Drop in DBP (mean ± SD, mmHg)	7.9 ± 4.5	7.5 ± 2.3	8.2 ± 5.3	0.56
UMSARS-1	15.9 ± 8.4	15.4 ± 5.0	16.2 ± 9.8	0.84
UMSARS-2	16 ± 8.2	16.8 ± 7.7	16.9 ± 9.6	0.92
UMSARS-4 (median; range)	2 (1–5)	2 (1–5)	2 (1–5)	-

MRI features

**Figure 1:**
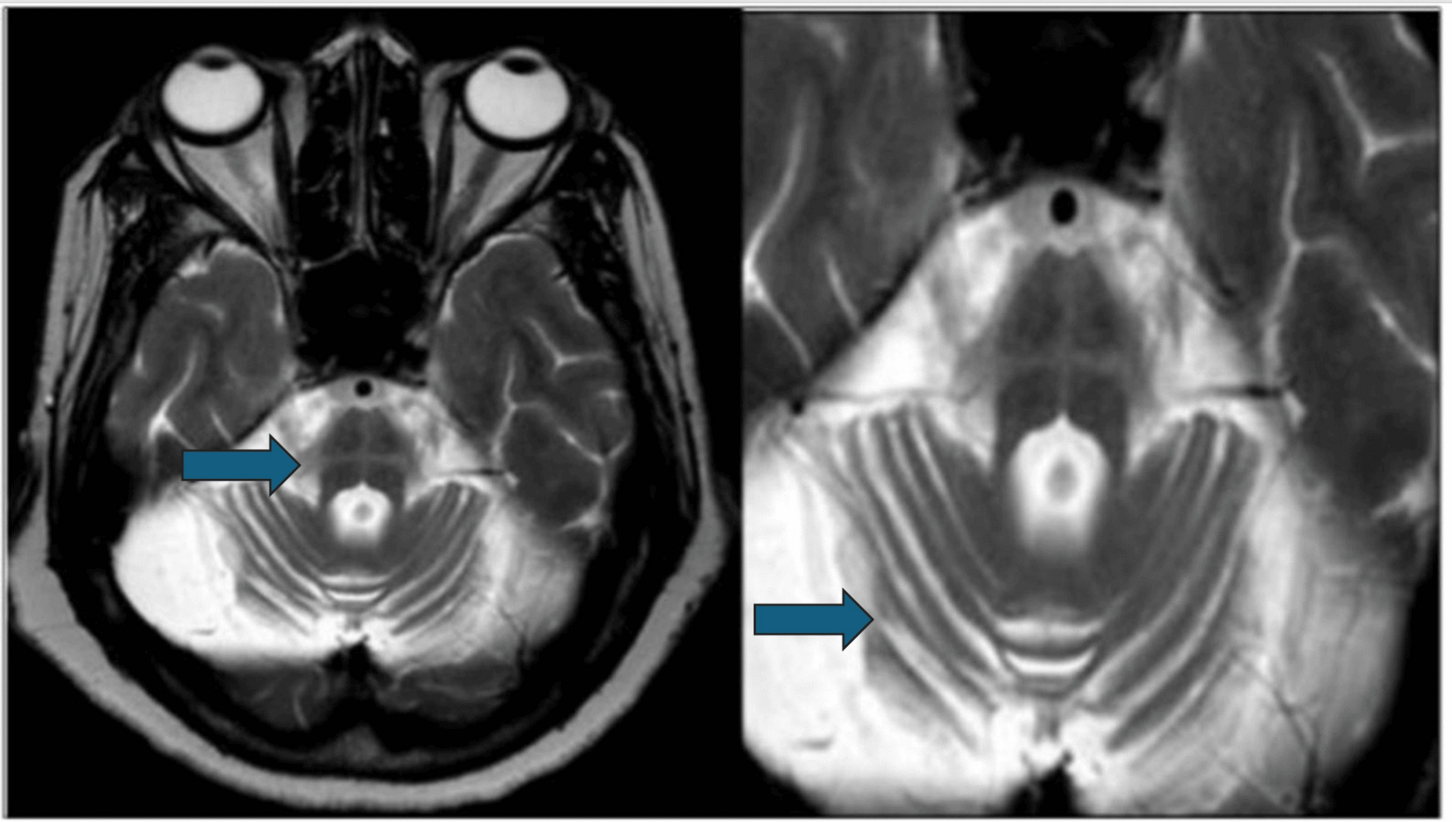
T2W axial images at the level of the superior cerebellar peduncle showing cruciate hyperintensity in the pons (hot cross bun sign) in MSA-C. The arrow in the first image is pointing toward cruciform hyperintensity in pons (hot cross bun sign). The arrow in the second image is pointing toward severe cerebellar atrophy in MSA-C. MSA-C = multiple system atrophy-cerebellar; T2W = T2-weighted

**Figure 2:**
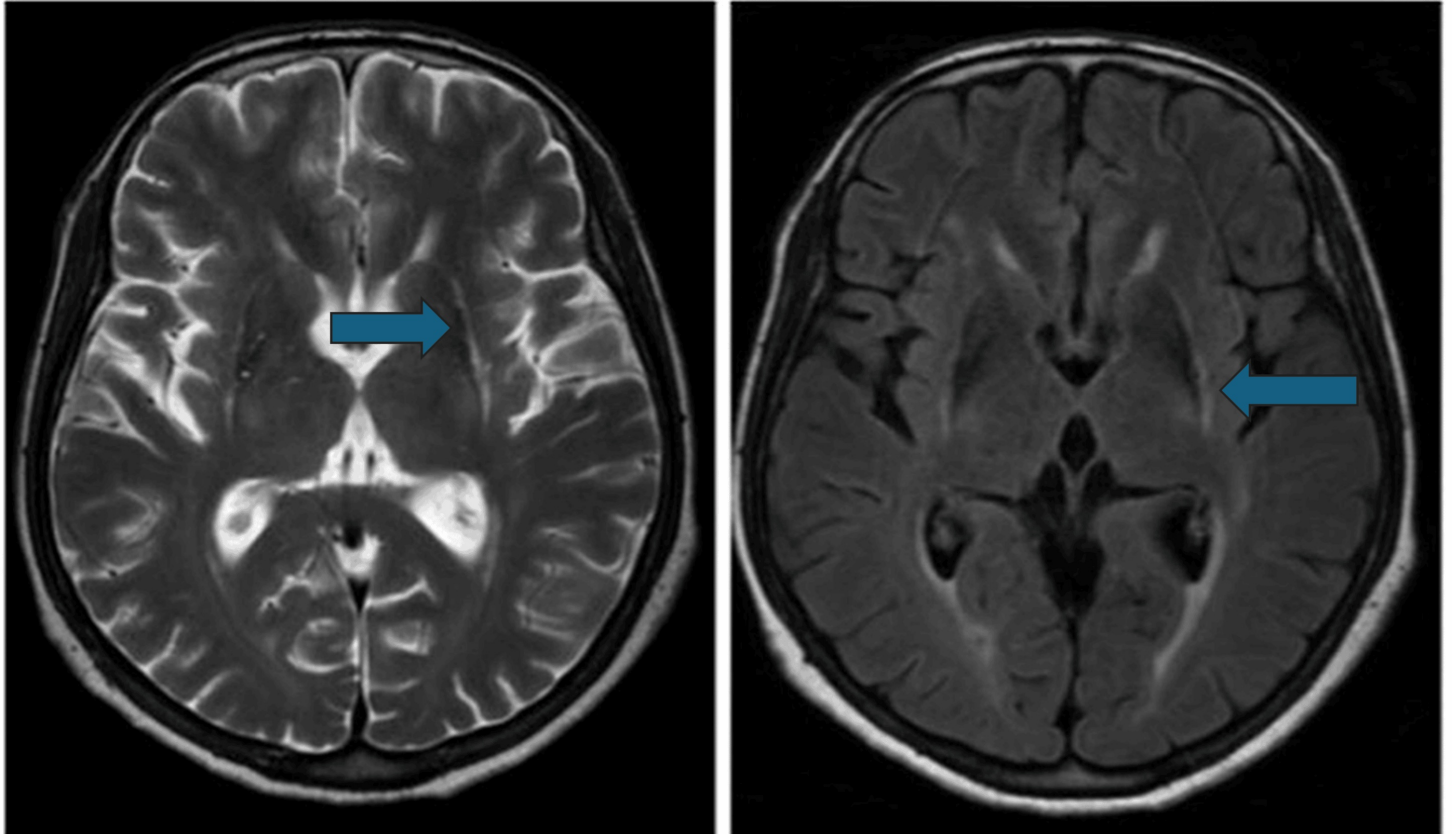
T2 and FLAIR axial images at the level of basal ganglia: a hyperintense rim along the lateral margin of the bilateral putamen (hyperintense putaminal rim sign) in MSA-P. Atrophy of the posterior aspect of the bilateral putamen is also noted. Arrows in the two images are pointing toward the hyperintensity of the bilateral putamen (putaminal rim sign). FLAIR = fluid-attenuated inversion recovery; MSA-P = multiple system atrophy-parkinsonian

**Figure 3:**
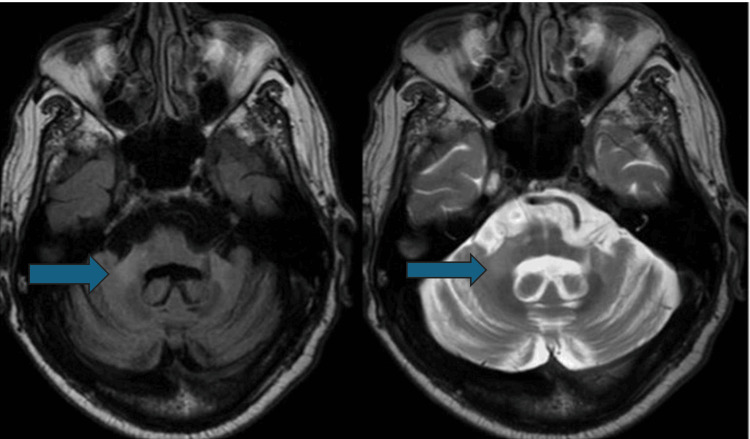
FLAIR and T2 axial images showing bilateral symmetrical hyperintensity of MCP (bright MCP sign) in MSA-C. Diffuse cerebellar atrophy is also noted. Arrows in these images are pointing toward the hyperintensity of the bilateral MCP (bright MCP sign). FLAIR = fluid-attenuated inversion recovery; MCP = middle cerebellar peduncle; MSA-C = multiple system atrophy-cerebellar

All patients had an abnormal brain MRI. Among 27 (90%) patients, cerebellar atrophy was the most frequently observed abnormality on MRI (MSA-C = 100%, MSA-P = 70%). The second most frequent MRI result, observed in 22 (73.3%) patients, was diffuse cerebral atrophy (MSA-P = 6 (60%), MSA-C = 16 (80%)). The hot cross bun (HCB) sign was seen in 15 (75%) MSA-C but none of the MSA-P patients (Figure 1). Seven patients had putaminal atrophy (MSA-C = 2 (10%) and MSA-P = 5 (50%)). Posterolateral putamen hyperintensity (putaminal rim sign) was seen in three (30%) MSA-P and two (10%) MSA-C patients (Figure 2). Nine patients had MCP hyperintensities (bright MCP sign). This bright MCP sign was present in 35% (7) of MSA-C patients and 20% (2) of MSA-P patients (Figure 3). All MRI features are shown in Table 2.

**Table 2:** MRI markers in MSA and its different subtypes. MCP = middle cerebellar peduncle; MSA = multiple system atrophy; MSA-P = multiple system atrophy-parkinsonian; MSA-C = multiple system atrophy-cerebellar

MRI markers	MSA (N = 30)	MSA-P (N = 10)	MSA-C (N = 20)
Cerebellar atrophy	27 (90%)	7 (70%)	20 (100%)
Diffuse cerebral atrophy	22 (73.3%)	6 (60%)	16 (80%)
Hot cross bun sign	15 (50%)	0	15 (75%)
Bright MCP sign	9 (30%)	2 (20%)	7 (35%)
Putaminal atrophy	7 (23%)	5 (50%)	2 (10%)
Putaminal rim sign	5 (16.6%)	3 (30%)	2 (10%)

Correlations of MRI features with disease severity

Using Spearman’s correlation coefficient, we found a strong (p < 0.05) correlation between the HCB sign and a higher UMSARS-4 score (global disability) in MSA-C patients (r = 0.14, p < 0.01). The mean duration of illness in patients with the HCB sign on MRI was 3.1 years, indicating a longer period of disease required to develop this sign on MRI in these patients. There was no discernible relationship between clinical severity and imaging markers in any of the MSA-P patients. This correlation is shown in Table 3.

**Table 3:** Correlation between imaging features and disease severity. P-values <0.05 are significant. MCP = middle cerebellar peduncle; MSA-P = multiple system atrophy-parkinsonian; MSA-C = multiple system atrophy-cerebellar; UMSARS = Unified Multiple System Atrophy Rating Scale

MRI feature	UMSARS-4 (MSA-P)		UMSARS-4 (MSA-C)	
	r	P-value	r	P-value
Hot cross bun sign	0.14	<0.01	Nil	Nil
Bright MCP sign	0.44	0.29	0.32	0.65
Putaminal atrophy	0.35	0.50	0.54	0.60
Putaminal rim	0.30	0.56	0.38	0.50

## Discussion

This cross-sectional study represents the largest cross-sectional study from South India, involving 30 clinically diagnosed MSA patients. The average age of symptom onset was 51.8 ± 5.9 years, with MSA-C exhibiting an earlier onset compared to MSA-P (51 ± 6.4 vs. 53.3 ± 4.7 years), consistent with prior research findings [8]. A higher prevalence in males was observed, mirroring a study from Japan [9]. The mean illness duration was 2.6 ± 1.2 years, with MSA-C cases showing a longer duration, contrary to other studies indicating durations of 15 years or more [10]. Our distribution of MSA subtypes, with a majority of MSA-C cases (67%), reflects trends in Asia, contrasting with European and North American studies where MSA-P is more prevalent, suggesting geographical or genetic influences [11-13]. Our study highlights geographical differences in MSA subtypes, with MSA-C more prevalent in Asia and not influenced by gender.

Gait ataxia was the most common symptom, seen in 43.3% of patients, closely matching previous reports [14]. Autonomic symptoms preceded motor symptoms in 13% of cases, lower than previous estimates [15]. The average UMSARS-2 score was 16 ± 8.2, consistent with prior studies [16]. Autonomic dysfunction, particularly orthostatic hypotension, was observed in 33.3% of our cohort, lower than other reports presenting a range from 43% to 81% [17,18]. Nonetheless, the severity of autonomic dysfunction between MSA-C and MSA-P groups was similar, affirming uniformity in autonomic involvement. Urinary dysfunction was the most common autonomic feature, affecting 96.7% of patients, with 65% reporting urinary urgency, in line with findings that emphasize its prevalence in MSA over Parkinson’s disease [19,20]. These clinicodemographic features highlight the variability in MSA presentation and the high prevalence of autonomic dysfunction among MSA patients.

Neuroimaging in multiple system atrophy

Our investigation into MSA encompassed MRI analysis of 30 individuals, confirming abnormalities in each case, yielding a detection accuracy of 100%. Predominantly, cerebellar atrophy was noted in 90% of the subjects, universally present in MSA-C (100%), and observed in 70% of MSA-P patients. This finding resonates with prior research which also noted cerebellar atrophy in all MSA-C and half of MSA-P patients [21]. This consistency underscores cerebellar atrophy as a hallmark MRI feature of MSA, particularly in MSA-C.

Second to cerebellar atrophy, diffuse cerebral atrophy was present in 73.3% of the cohort, with a higher occurrence in MSA-C (80%) than in MSA-P (60%) patients. This aligns with previous study reports where significant cerebral atrophy was seen in MSA patients when compared to healthy controls over a year [22]. The HCB sign, which signals degeneration of the pontocerebellar tracts, was observed in half of all MSA cases, mainly in MSA-C (75%), but was absent in MSA-P. This finding contrasts with other studies where the HCB sign was noted in 63% of MSA cases, including 77% in MSA-P. This discrepancy could be attributed to the shorter duration of illness in our study group, which may influence the emergence of the HCB sign [23,24].

Atrophy and signal changes in the posterior putamen, namely, the putaminal rim sign, were detected in 23% and 16.6% of patients, respectively, mostly linked to MSA-P. Specifically, half of MSA-P and only 10% of MSA-C patients showed putaminal atrophy, with 30% of MSA-P and 10% of MSA-C patients exhibiting the putaminal rim sign [25]. Notably, the rim sign can appear in normal MRIs with a 3T machine, suggesting it is not unique to MSA-P [26]. Our findings show a lower prevalence of these putaminal abnormalities, likely due to our stringent bilateral criteria, unlike previous analyses that considered unilateral changes [27].

The bright MCP sign was observed in 30% of patients, with a higher frequency in MSA-C (35%) compared to MSA-P (20%), contrasting with a study reporting an 87% prevalence in MSA [28]. Spearman’s analysis revealed a significant correlation between the HCB sign and disease severity in MSA-C patients, supported by previous findings linking the HCB sign with impairment scores in MSA-C [29]. However, no such correlation was found for MSA-P, aligning with reports of no significant link between the HCB sign and disease severity in MSA-P [30].

Limitations

Our study’s limitations include the short illness duration of participants, potential referral bias from the tertiary center setting, stringent imaging criteria, non-blinding of neuroradiologists, and no correlation with HCB grade. These factors may impact the generalizability of our findings, emphasizing the need for caution in interpretation and the importance of addressing them in future research.

## Conclusions

This study highlights the high prevalence of cerebellar atrophy in patients with MSA-C, which is strongly associated with disease severity, underscoring the importance of specific imaging markers in diagnosing and assessing this subtype. Conversely, no association was found between imaging markers and disease severity in MSA-P.
